# Coronavirus pandemic impact on bank performance

**DOI:** 10.3389/fpsyg.2022.1014009

**Published:** 2022-10-06

**Authors:** Xing Xiazi, Mohsin Shabir

**Affiliations:** ^1^School of Economics, Shandong University of Finance and Economics (SDUFE), Jinan, Shandong, China; ^2^Youth League Committee of Shandong University of Finance and Economics, (SDUFE), Jinan, Shandong, China; ^3^School of International Trade and Economics, Shandong University of Finance and Economics (SDUFE), Jinan, Shandong, China

**Keywords:** COVID-19 pandemic, bank performance, institutional quality, financial development, global banking

## Abstract

This study examines the effects of the coronavirus (COVID-19) epidemic on the performance of the banking sector. Our sample consists of 1,575 banks in 85 countries from 2020Q1 to 2021Q4. The findings demonstrate that the COVID-19 outbreak has significantly decreased bank performance. Moreover, the adverse impact of COVID-19 on the bank’s performance depends on the bank’s and country-specific aspects. The adverse effect of the COVID-19 outbreak on bank performance is higher in smaller, undercapitalized, and less diversified banks. At the same time, a better institutional environment and financial development have significantly increased the strength and resilience of banks. The results are quite robust to using the alternative bank performance measures and estimation techniques. These findings provide practical implications for regulators and policymakers in the face of unprecedented uncertainty caused by COVID-19 epidemics.

**JEL Classification:** G01, G21, L50.

## Introduction

In early December 2019, Wuhan City, China, first observed the beginning of the novel coronavirus (COVID-19; Demir and Danisman, 2021; Padhan and Prabheesh, 2021). Since its origin in Wuhan, COVID-19 has wreaked havoc worldwide because it is a highly transmittable and pathogenic viral infection (Zaremba et al., 2020). Countries including China, Italy, Spain, France, the United Kingdom, and the United States have been hit hard by the severe COVID-19 outbreak (Padhan and Prabheesh, 2021; Song et al., 2021). Therefore, on March 11, 2020, the World Health Organization (WHO) stated that COVID-19 is a global pandemic outbreak and is considered a “once-in-a-century pathogen” for the following reasons. Firstly, COVID-19 is perhaps a unique outcome in terms of its global scope as a pandemic. This disease’s rapid transmission rate shows that COVID-19 is more dangerous than any other pandemic (Padhan and Prabheesh, 2021). Secondly, the mortality risk of COVID-19 is 1%, which is worse than normal influenza because it can kill healthy and older people. This fatality risk is comparable to the 1,857 influenza pandemic (0.6%) and the 1,918 Spanish flu (2%). However, due to the absence of pharmaceutical innovations, the actual death rate of COVID-19 is unpredictable (Padhan and Prabheesh, 2021).

The outbreak of COVID-19 brought seriously affected health care, economy, transportation, and other fields in different industries and regions (Shen et al., 2020). Moreover, the COVID-19 pandemic has reverberated across economies and financial markets and greatly impacted real economic activity. The economic impact of COVID-19 can be generally divided into two aspects: supply and demand impacts. The supply impact is the outcome of reductions in working hours and aggregate demand resulting from reduced incomes due to unemployment related to the lockdown. However, Maliszewska et al. (2020) highlight the four key channels through which a pandemic affects the economy. First, it directly impacts through the decrease in employment, which drives a reduction in the demand for capital, leading to a loss in output. Second, the rise in transaction costs increases the costs of imports and exports for goods and services, resulting in a drop in trade and productivity. Third, with a sharp reduction in travel, governments have imposed several restrictions to reduce infections that disrupt international tourism and lead to lower incomes and loss of productivity. Finally, the declined in demand for services. The demand seems to have taken a big hit, as these emergency shutdowns have also locked households into their homes, dramatically reducing consumer spending.

However, this ongoing COVID-19 has taken significant losses for countless businesses, leading to serious disruptions in various industries (Khlystova et al., 2022). A growing body of literature observes the potential impact of the COVID-19 pandemic on different aspects. In this regard, one group of researchers focuses on the short-term effect of COVID-19 on stock market returns or volatility. They show that COVID-19 significantly reduces stock market returns and increases stock market volatility (Al-Awadhi et al., 2020; Baek et al., 2020; Zaremba et al., 2020; Harjoto et al., 2021). At the same time, the other group of researchers has investigated the COVID-19 pandemic’s impact on the firm’s financial performance in various sectors (Shen et al., 2020; Hu and Zhang, 2021; Atayah et al., 2022; Wellalage et al., 2022). Shen et al. (2020) show that firm performance worsens during the COVID-19 pandemic, which is more significant when a firm’s sales revenue or investment scale is low. At the same time, Hu and Zhang (2021) reported that the adverse effect of COVID-19 on firm performance is less pronounced in countries with better institutional environments, well-developed financial systems, and better health care systems.

While this unexpected shock is likely to impact banks, little is yet known about how it might affect the resilience and performance of the banking system as a whole (Goodell, 2020; Duan et al., 2021). This is because the bank generally faces a broader range of risks compared to other financial institutions and is more closely connected with economic agents’ day-to-day activities (Barua and Barua, 2020). Banks traditionally deal with a wide range of risks. The pandemic is set to exacerbate them through liquidity shortages, credit reduction, falling returns from investments, and rises in non-performing loans and default rates (Barua and Barua, 2020; Goodell, 2020). This may be worse in nations where banks support millions of individuals and firms with comparatively low financial and economic capacity under a weak policy environment and high market competition (Barua and Barua, 2020).

Coronavirus can affect banks in different ways. For example, banks worldwide hold large US dollar-denominated borrowings to fund international trade and financial investments (Aldasoro and Ehlers, 2018). Financial crises tighten the money markets that lend dollars, implying risks for the global banking system. However, as a first response to the pandemic, central banks stretched current swap lines and formed new lines to reduce the cost of dollar funding (Bahaj and Reis, 2020; Demir and Danisman, 2021). Bank prudential regulatory actions, such as relaxing the treatment of non-performing loans and reducing capital buffers, mitigate the adverse effect of COVID-19 on the financial system’s stability (Demir and Danisman, 2021; Bitar and Tarazi, 2022). Danisman et al. (2021) presented that equity markets in countries with more strict regulatory requirements on capital and liquidity are more resilient to COVID-19. However, due to Basel III capital and liquidity reforms since 2008, banks are well situated to engage the extreme effect of COVID-19. At the same time, facilitation of the behavior of non-performing loans and capital buffers during a pandemic can put banks’ solvency at risk. The possibility of an increase in non-performing loans and substantial withdrawal of deposits by firms and households will adversely affect the performance of banks (Danisman et al., 2021; Goodell, 2020). Besides, COVID-19 could adversely affect the efficiency of firms across all businesses, and there could be spillover effects on banks, which would increase their exposure to credit risk. This would threaten their stability and create some obstacles to future intermediation with some potential spillovers to the real economy (Demir and Danisman, 2021).

This study contributes to the literature in the following aspects. First, prior literature examined the impact of COVID-19 on macroeconomic prospectives, such as economic growth (Apergis and Apergis, 2021), International Trade (Gruszczynski, 2020; Vidya and Prabheesh, 2020), oil price (Mensi et al., 2020; Gharib et al., 2021), and gold price (Mensi et al., 2020). While at the firm level, most existing studies focus on the impact of COVID-19 on a firm’s performance (Fu and Shen, 2020; Gu et al., 2020; Shen et al., 2020; Xiong et al., 2020; Škare et al., 2021) and stock market returns and volatility (Al-Awadhi et al., 2020; Baek et al., 2020; Zaremba et al., 2020; Harjoto et al., 2021). At the same time, the impact of COVID-19 on bank performance is scarce. In this study, we shifted the perspective to bank performance at the international level. Secondly, we explored the mechanisms through which COVID-19 affects bank performance. Lastly, as COVID-19 spreads globally, we determine whether the impact of COVID-19 on bank performance varies with institutional quality and level of financial development.

This study examines how the COVID-19 outbreak impacts the banking sector’s performance worldwide. Our sample comprises 1,575 listed and unlisted banks in 85 countries from 2020Q1 to 2021Q4. We use numerous alternative bank performance measures for a comprehensive examination and robustness. The findings illustrate that COVID-19 has significantly decreased bank performance. Moreover, the COVID-19 epidemic’s effects on the bank’s performance vary in the bank’s and country-specific aspects. The finding shows that adverse effects of COVID-19 on bank performance are more pronounced in smaller, undercapitalized, and less diversified banks. Also, a better institutional environment and financial development diminish the negative effects of COVID-19 on banks’ performance. Our primary results continue across alternative model specifications (i.e., GMM).

The rest of the paper is organized as follows. The section “Literature review” provides an overview of the relevant literature. The section “Data and methodology” defines our sample, study variables, econometric model, and summary statistics. The sections “Results and discussion” and “Robustness checks” report the empirical outcomes and robustness tests, respectively. The section “Conclusion” presents the conclusion of the paper.

## Literature review

Coronavirus is a major health emergency worldwide. The outbreak of COVID-19 has severely affected healthcare, economy, transportation, and other sectors in various industries and regions (Padhan and Prabheesh, 2021). This increasing pandemic in emerging and developed countries has led to strict lockdowns and unprecedented economic activity disruptions (Baldwin and di Mauro, 2020; Padhan and Prabheesh, 2021). For example, in the second quarter of 2020, global GDP fell by more than 4.9 percent due to economic disruption (Padhan and Prabheesh, 2021). The deterioration in the international trade in goods and services was possibly greater than during the global financial crisis of 2007–08 (IMFC, 2020). Therefore, due to weak supply and demand, international trade was constricted by 3.5 percent in the second quarter of 2020 (Vidya and Prabheesh, 2020). A rapid drop in consumption of goods and services was observed due to a sharp drop in income and weak consumer confidence. Moreover, emerging countries experienced substantial capital outflows and reduced investment and productivity due to the pandemic (BIS, 2019; Padhan and Prabheesh, 2021).

Moreover, prior literature analyzed the COVID-19 impact in several ways. For example, Choi (2020) and Njindan Iyke (2020) stated that due to COVID-19, production and credit were reduced. Bauer and Weber (2020), Liu et al. (2020), and Yu et al. (2020) proved a significant reduction in consumption, investment, and labor force participation rate. Moreover, COVID-19 has unfavorably affected corporate performance (Gu et al., 2020; Shen et al., 2020) and herding behavior (Espinosa-Méndez and Arias, 2020). Additionally, some researchers have explored the impact of COVID-19 on the price of oil (Fu and Shen, 2020; Narayan, 2020). They highlighted that the deterioration in oil prices due to the pandemic unfavorably influenced the energy sector’s performance. Fu and Shen (2020) and Narayan (2020) argued that COVID-19 increased oil price volatility and negatively affected energy industries,

The COVID-19 pandemic also amplified worldwide financial risks and destructively affected international financial markets (Al-Awadhi et al., 2020; Cao et al., 2020; Harjoto et al., 2020). COVID-19 has adversely impacted the stock market in the form of ambiguity and a decline in global stock returns, decreasing capital inflows, and creating constraints on investment, new project financing, and accessibility to liquidity in the international financial system (Padhan and Prabheesh, 2021). Guedhami et al. (2021) found that international firms experienced considerably lower stock prices than domestic companies during the pandemic crisis. They also demonstrate that the better financial system of the country moderates these negative performance impacts while real characteristics increase negative crisis returns. Al-Awadhi et al. (2020) and Wang and Enilov (2020) show that COVID-19 significantly reduces stock market returns. Zaremba et al. (2020) illustrate that COVID-19 led to a significant rise in stock market volatility.

Additionally, COVID-19 has a devastating effect on the efficiency of firms across all businesses and may have spillover effects on banks, increasing their exposure to credit risk. Acharya and Steffen (2020) revealed that the increased pace of reducing credit growth, particularly by riskier companies, could damage bank balance sheets and lesser their capital adequacy ratios. This would threaten their stability and create obstacles to upcoming intermediation by possible spillovers to the real economy. Li et al. (2020) reported that U.S. banks significantly amplified their lending if they had more idle loan commitments at the start of the pandemic. However, they argued that though banks improved their credit growth, their total credit supply remained unchanged. In the same way, Greenwald et al. (2020) show that U.S. banks that experienced large credit line drawdowns were more restrictive in lending to small firms during the COVID-19 crisis. Beck and Keil (2021) demonstrate that U.S. banks faced increasing loan loss provisions and non-performing loans. Hasan et al. (2021) illustrate that the spread of syndicated loans increased as the lender or borrower became more vulnerable to epidemics. Ҫolak and Öztekin (2021) and Duan et al. (2021) investigate the effect of the pandemic on global lending and banks’ systemic risk from an international perspective. Demir and Danisman (2021) show that stock returns of banks with a large size, lesser non-performing loans, well capitalization, and higher deposits are more resilient to the pandemic. Dursun-de Neef et al. (2022) show that worse-capitalized banks increased their loan supply significantly more during the pandemic. Elnahass et al. (2021) show that COVID-19 significantly affects financial performance over various financial performance and stability measures. Demirgüç-Kunt et al. (2021) argued that liquidity assistance, borrower support programs, and monetary easing moderated the negative effects of the crisis, but their effects varied significantly across banks and countries. Therefore, based on this evidence in this study, we will find the impact of COVID-19 on bank performance.

## Data and methodology

### Data and sample selection

To analyze the impact of COVID-19 on the banking sector, we obtained quarterly balance sheet data of 1,575 listed and unlisted banks in 85 different countries from the Bankscope database for 2020Q1 to 2021Q4.^1^ Quarterly frequency data are preferred for the following reasons: (a) The most important reason is that daily and monthly data are unavailable for financial and accounting data; (b) the COVID-19 period covers only two quarters. Hence, our frequency is driven by current financial and accounting data availability in 2020–21. Country-related variables such as GDP *per capita*, inflation, and bank concentration are taken from IMF and World Bank. Table 1 reports a detailed explanation of all variables and sources. Table 2 displays the summary statistics of the variables of interest.

**Table 1:** Variables definition.

Variable	Definition	Source
**Dependent variables**		
Return on average assets (ROAA)	Net income scaled by average total assets	Bank scope
Return on average equity (ROAE)	Net income scaled by average total equity	Bank scope
**Explanatory variables**		
Covid-19 (COVID-19)	The total number of COVID-19 confirmed cases per million in the country	
Size (SIZ)	Natural logarithm of bank assets	Bank scope
Capital (CAP)	Equity over total assets	Bank scope
Diversification (DIV)	Net non-interest income to net operating income ratio	Bank scope
Loan Share (LTA)	Net loan to total assets	Bank scope
Liquidity (LIQ)	Liquid assets divided by total assets	Bank scope
GDP *per capita* (GDPpc)	Natural logarithm of GDP *per capita*	IFS Data
Inflation (INF)	Inflation based on the CPI	IFS Data
Concentration ratio (CON)	Percentage of the five largest bank assets to the country’s total bank assets.	GFDD

This table presents detailed descriptions of study variables.

**Table 2:** Descriptive statistics.

Variable	Obs	Mean	SD	Min	Max
ROAA	2,489	9.288	15.592	−14.731	33.087
ROAE	2,489	9.262	13.981	−44.731	77.256
NIM	2,554	4.371	4.228	−1.656	24.474
CIN	2,554	63.439	29.52	6.314	215.874
NPL	1,202	1.030	1.455	−12.276	3.929
COVID-19	45,606	0.136	0.343	0	1
SIZ	2,800	7.543	2.664	1.879	13.766
CAP	2,796	15.745	14.578	5.016	82.052
LIQ	2,800	25.437	17.742	15.947	71.305
LTA	2,726	58.825	20.014	0.381	92.781
DIV	2,575	36.784	27.988	−27.427	121.392
CON	3,098	56.466	15.633	17.164	91.106
GDPpc	3,148	1.442	4.300	−11.234	7.521
INF	3,081	2.860	2.797	−1.247	19.628

This table shows summary statistics for the variables used in this study.

### Measurements of variables

#### Bank performance measurement

Although banking institutions have become gradually complex, profitability is the underlying driver of bank performance. In this study, we used the two accounting-based measures that are widely used in the earlier literature (Adesina, 2021; Dang and Dang, 2021; Elnahass et al., 2021) as a dependent variable to evaluate the bank’s performance. These accounting-based measures return on average total assets (ROAA) and return on average equity (ROAE). These are the banking sector’s most accepted financial performance measures (Simpson and Kohers, 2002). Moreover, we also used several alternative proxies of bank performance as robustness.

#### COVID-19 indicators

In this study, we follow Ҫolak and Öztekin (2021) and use the total number of COVID-19 confirmed cases per million in the country as a proxy for COVID-19.

#### Bank and country-specific variables

In addition to COVID-19, we have incorporated numerous bank-related and country-related control variables in our model to address the potential omitted variables problem. The bank-related control variables are bank size (SIZ), capitalization (CAP), liquidity (LIQ), asset structure (LTA), and bank diversification (DIV). Bank size (SIZE) is measured through the natural logarithm of a bank’s total assets. Capitalization (CAP) is estimated as equity to total assets. Bank liquidity (LIQ) is calculated as the ratio of liquidity assets to total assets. Net loan to total assets (LTA) is used as the bank’s asset structure proxy. Bank diversification (DIV) is measured by the non-interest income ratio to net operating income. At the same time, the country-related control variables are GDP *per capita* (GDPpc), inflation (INF) and bank concentration (CON). We use GDP *per capita* and inflation rates to control business cycles’ overall effects, unobserved factors that vary across countries (Wu et al., 2020). Finally, bank Concentration (CON) controls the country’s market structure.

### Empirical framework

In this study, to examine the impact of the COVID-19 pandemic on bank performance, our baseline model is as follows


(1) $$BP_{ijt}=\beta_{1}COVID19_{t}+\gamma_{l}X_{it}+\delta_{k}Z_{jt}+\mu_{i}+\lambda_{t}+\varepsilon_{it}$$

where *I*, *j t* indicate the bank, country and quarter (time). BP denotes our dependent variables bank performance, which is measured as ROAA and ROAE. COVID-19 is our primary explanatory variable measured as the total number of COVID-19 confirmed cases per million in the country. $X_{it}$ is a vector of our bank-related control variables. $Z_{jt}$ is a vector of country and market structure control variables. β, γ,andδ are the parameters of the model. Moreover, $\mu_{i}$ , and ʎt are the bank and time effects and $\varepsilon_{it}$ is the error term. We estimate equations (1) with the fixed-effects model.^2^

## Results and discussion

### Baseline regression results

Our core objective of the study is to determine the possible impacts of the COVID-19 pandemic on bank performance across countries. For this purpose, we regresses the bank performance on COVID19 and show our baseline regression model results in Table 3. In columns (1) and (5) in Table 3, we analyze the impact of COVID-19 on bank performance along cross-sectional and time fixed-effects, but we do not incorporate bank and country-related control variables. In columns (2) and (6), we contain the bank-related control variable, while in columns (3) and (7); we comprise country-related control variables. In columns (4) and (8), we incorporate all bank and country-related control variables with cross-sectional and time fixed-effects to examine the impacts of COVID-19 on bank performance. Overall, our findings highlight the significant negative effects of COVID-19 on bank performance in the sampling countries. Columns (4) and (8) in Table 3 show that COVID-19 coefficients are statistically significant with a negative sign with both ROAA and ROAE of bank performance measures. This finding is consistent with Elnahass et al. (2021) and shows that the outbreak of COVID-19 has significantly decreased the banking sector’s profitability. To simplify the economic interpretation of the regression coefficients of our key variables of interest, we implement a log transformation to COVID-19. The coefficient of COVID-19 reflects the βCOVID-19% change in bank performance for a 1% change in the number of disease cases per million.

**Table 3:** COVID-19 impact on bank performance.

	(1)	(2)	(3)	(4)	(5)	(6)	(7)	(8)
Variables	ROAA	ROAA	ROAA	ROAA	ROAE	ROAE	ROAE	ROAE
COVID-19	−0.126^*^ (0.070)	−0.330^***^ (0.070)	−0.225^***^ (0.085)	−0.383^***^ (0.085)	−1.544^***^ (0.389)	−2.185^***^ (0.398)	−0.577 (0.445)	−1.609^***^ (0.454)
SIZ		0.417^***^ (0.041)		0.530^***^ (0.066)		1.466^***^ (0.237)		3.352^***^ (0.361)
CAP		0.065^*^^**^ (0.002)		0.078^***^ (0.003)		0.030^**^ (0.015)		0.220^***^ (0.019)
LA		−0.179 (0.193)		0.021 (0.219)		1.216 (1.108)		0.338 (1.178)
LTA		0.009^***^ (0.002)		0.004^*^ (0.002)		0.106^***^ (0.011)		0.019^***^ (0.000)
DIV		0.016^***^ (0.000)		0.012^***^ (0.000)		0.079^***^ (0.004)		0.070^***^ (0.005)
CON			0.010^***^ (0.003)	0.010^***^ (0.003)			0.093^***^ (0.016)	0.096^***^ (0.016)
GDPpc			0.008 (0.007)	0.012^*^ (0.007)			0.227^***^ (0.037)	0.237^***^ (0.037)
INF			−0.031^***^ (0.007)	−0.021^***^ (0.007)			−0.052 (0.040)	0.310^***^ (0.040)
$\alpha_{{}_{0}}$	1.065^***^ (0.053)	−4.095^***^ (0.374)	0.560^***^ (0.180)	−5.299^***^ (0.598)	9.241^***^ (0.296)	−11.41^***^ (2.151)	3.410^***^ (0.932)	−28.87^***^ (3.250)
Observations	2,489	2,424	2,407	2,345	2,489	2,424	2,407	2,345
R-squared	0.013	0.051	0.023	0.051	0.013	0.028	0.026	0.042
Bank FE	Yes	Yes	Yes	Yes	Yes	Yes	Yes	Yes
Time FE	Yes	Yes	Yes	Yes	Yes	Yes	Yes	Yes

This table shows the results of the baseline regression on analyzing the effect of the COVID-19 epidemic on bank performance. The sample comprises 1,575 banks in 85 countries from 2020 Q1 to 2021 Q4. Our dependent variable is bank performance measured as ROA and ROE. COVID-19 is our primary explanatory variable of interest, measured as the total number of COVID-19 confirmed cases per million in the country. Robust standard errors are reported in parentheses.

*** *p* < 0.01.

** *p* < 0.05.

* *p* < 0.1.

This finding can be interpreted as the spread of the virus forcing governments to initiate several preventive measures, such as social distancing, lockdowns, and business shutdowns (Duan et al., 2021). These activities, in turn, lead to adverse economic impacts on firms and households. As a result, firms have experienced significant declines in revenue and increased cost, and households have experienced job losses and income declines (Duan et al., 2021). Therefore, firms and households may not be able to service their debt, increasing the probability of default (Bartik et al., 2020). These effects are likely to spread to banks, resulting in lost revenue and a surge in non-performing loans, negatively affecting banks’ profits, capital, and solvency (Beck and Keil, 2021). Furthermore, lower demand for bank services may result in lower non-interest income, lowering bank profitability and performance (Beck and Keil, 2021).

Regarding the first set (bank-specific) of control variables, we find that the bank size (SIZE) coefficients are statistically significant and positively linked with ROAA and ROAE. This result aligns with earlier studies by Adesina (2021) and Dang and Dang (2021) and shows that large banks have high ROAA and ROAE. Similarly, capitalization (CAP) also significantly positive impacts ROAA and ROAE. These outcomes supported the empirical finding of Chortareas et al. (2012) and Adesina (2021), suggesting that better-capitalized banks are highly efficient than those with a lower capital base. Also, the coefficients of asset structure (LTA) significantly positively impact ROAA and ROAE, demonstrating that a better bank asset structure enhances bank profitability. Lastly, bank diversification is also positively associated with ROAA and ROAE. These results support the bank’s diversification advantage and show that reliance on sources of non-interest income enhances the bank’s profits.

In contrast, regarding the country-related control variables. This outcome indicates greater concentration enhances the banking sector’s performance and efficiency. The GDP *per capita* coefficients show a significant positive relationship with bank performance. At the same time, the estimated inflation coefficients show an adverse and highly significant relationship in all bank performance measures. The bank concentration coefficient is positively linked with ROAA and ROAE.

### Bank heterogeneity

Furthermore, we extend our basic analysis to examine how bank characteristics shape the effects of COVID-19 shocks on bank performance. Current studies have shown that bank features such as size, capitalization, liquidity, and diversification have significantly influenced bank performance (Altunbas et al., 2012; Shabir et al., 2021). Therefore, to estimate the heterogeneity across the bank, we include the interaction terms of bank size, capitalization, liquidity, and diversification with COIVD-19 in our main regression model. The results are reported in Table 4. The outcomes show that the coefficients on the interaction term of COVID-19 with size, liquidity, and diversification are positive and statistically significant on ROAA and ROAE. At the same time, the coefficient of interactions of COVID-19 with capitalization is significantly negative with all bank performance measures in ROAA and ROAE. These findings indicate that large size, more liquid and well-diversified banks reduce the adverse impact of COVID-19 on bank performance. In contrast, the poorly capitalized bank increases the adverse impact of COVID-19 on bank performance.

**Table 4:** Role of bank heterogeneity.

	(1)	(2)	(3)	(4)	(5)	(6)	(7)	(8)
Variables	ROAA	ROAA	ROAA	ROAA	ROAE	ROAE	ROAE	ROAE
COVID-19	−1.078^***^ (0.120)	0.191^**^ (0.092)	−0.330^***^ (0.125)	−0.486^***^ (0.095)	−1.205^*^ (0.642)	−1.160^**^ (0.498)	−1.098 (0.668)	−2.984^***^ (0.507)
SIZ	0.479^***^ (0.066)	0.448^***^(0.066)	0.530^***^ (0.066)	0.534^***^ (0.066)	3.384^***^ (0.363)	3.279^***^ (0.362)	3.357^***^ (0.361)	3.414^***^ (0.361)
CAP	0.077^***^ (0.003)	0.082^***^ (0.003)	0.078^***^ (0.003)	0.078^***^ (0.003)	0.221^***^ (0.019)	0.222^***^ (0.019)	0.220^***^ (0.019)	0.224^***^ (0.019)
LA	0.035 (0.218)	0.081 (0.217)	0.023 (0.219)	0.036 (0.219)	0.329 (1.178)	0.384 (1.178)	0.367 (1.178)	0.538 (1.177)
LTA	0.004^*^ (0.002)	0.004^*^ (0.002)	0.004^*^ (0.002)	0.004^*^ (0.002)	0.010 (0.012)	0.011 (0.012)	0.013 (0.012)	0.013 (0.012)
DIV	0.013^***^ (0.000)	0.012^***^ (0.000)	0.012^***^ (0.000)	0.011^***^ (0.000)	0.070^***^ (0.005)	0.071^***^ (0.005)	0.070^***^ (0.005)	0.061^***^ (0.005)
CON	0.012^***^ (0.003)	0.014^***^ (0.003)	0.010^***^ (0.003)	0.010^***^ (0.003)	0.095^***^ (0.016)	0.098^***^ (0.016)	0.096^***^ (0.016)	0.095^***^ (0.016)
GDPpc	0.010 (0.007)	0.022^***^ (0.007)	0.012^*^ (0.007)	0.012^*^ (0.007)	0.238^***^ (0.037)	0.245^***^ (0.037)	0.239^***^ (0.037)	0.238^***^ (0.037)
INF	−0.027^***^ (0.007)	−-0.027^***^ (0.007)	−0.020^***^ (0.007)	−0.020^***^ (0.007)	0.014 (0.040)	0.006 (0.040)	0.013 (0.041)	0.024 (0.040)
SIZ^*^COVID-19	0.090^***^ (0.011)				0.184^***^ (0.071)			
CAP^*^COVID-19		−0.035^***^ (0.002)				−0.027^**^ (0.012)		
LIQ ^*^COVID-19				0.054^**^ (0.026)				0.015^***^ (0.002)	
DIV^*^COVID-19				0.00294^**^ (0.122)				0.039^***^ (0.006)
$\alpha_{{}_{0}}$	−5.018^***^ (0.598)	−4.902^***^ (0.596)	−5.320^***^ (0.599)	−5.328^***^ (0.598)	−29.05^***^ (3.257)	−28.48^***^ (3.255)	−29.09^***^ (3.257)	−29.27^***^ (3.248)
Observations	2,345	2,345	2,345	2,345	2,345	2,345	2,345	2,345
*R*-squared	0.054	0.061	0.051	0.051	0.042	0.042	0.042	0.043
Bank FE	Yes	Yes	Yes	Yes	Yes	Yes	Yes	Yes
Time FE	Yes	Yes	Yes	Yes	Yes	Yes	Yes	Yes

This table demonstrates how a bank with diverse characteristics responds to the COVID-19 epidemic. The sample comprises 1,575 banks in 85 countries from 2020 Q1 to 2021 Q4. Our dependent variable is bank performance measured as ROA and ROE. COVID-19 is our primary explanatory variable of interest, measured as the total number of COVID-19 confirmed cases per million in the country. Robust standard errors are reported in parentheses.

*** *p* < 0.01.

** *p* < 0.05.

* *p* < 0.1.

### COVID-19 and bank performance: Role of the institutional quality

The quality of institutions plays an important role during the financial crisis (Klomp and De Haan, 2014; Fazio et al., 2018). Numerous recent studies have shown that various aspects of the formal and informal institutional environment significantly affect a bank’s profitability and risk levels. Beck et al. (2006) show that the regulatory policies and institution quality are significantly related to the banking system’s stability. Klomp and De Haan (2014) highlight that tight regulatory policy and higher supervision power reduce bank risk. To capture the institutional quality, we followed the previous literature and used Worldwide Governance Indicators (WGI), which contains six different aspects of institutional quality (i.e., government effectiveness, political stability, regulatory quality, control of corruption, the rule of law, and accountability). Therefore, for a more comprehensive analysis of COVID-19 and bank performance nexus, we include the interaction terms of COVID-19 with institutional quality in the regression model (1). The results are presented in Table 5, which shows that the coefficients of the institutional quality variable and their interaction terms with COVID-19 are significantly positive at different levels. This indicates that better institutional quality increases the bank’s performance in response to COVID-19 epidemics.

**Table 5:** Bank performance during the COVID-19 pandemic. The role of the institutional quality.

	(1)	(2)	(3)	(4)	(5)	(6)	(7)	(8)	(9)	(10)	(11)	(12)
Variables	ROAA	ROAA	ROAA	ROAA	ROAA	ROAA	ROAE	ROAE	ROAE	ROAE	ROAE	ROAA
COVID-19	−0.468^***^ (0.861)	−0.354^***^ (0.857)	−0.394^***^ (0.858)	−0.357^***^ (0.856)	−0.382^***^ (0.854)	−0.277^***^ (0.874)	−1.715^***^ (0.458)	−1.777^***^ (0.457)	−1.518^***^ (0.457)	−1.394^***^ (0.456)	−1.486^***^ (0.455)	−0.277^***^ (0.874)
SIZ	0.542^***^ (0.665)	0.508^***^ (0.667)	0.524^***^ (0.666)	0.511^***^ (0.666)	0.516^***^ (0.667)	0.506^***^ (0.667)	3.418^***^ (0.360)	3.420^***^ (0.362)	3.396^***^ (0.361)	3.406^***^ (0.361)	3.466^***^ (0.362)	0.506^***^ (0.667)
CAP	0.775^***^ (0.349)	0.770^***^ (0.350)	0.780^***^ (0.349)	0.773^***^ (0.349)	0.0779^***^ (0.350)	0.772^***^ (0.350)	0.214^***^ (0.192)	0.222^***^ (0.193)	0.220^***^ (0.0193)	0.219^***^ (0.193)	0.225^***^ (0.193)	0.772^***^ (0.350)
LA	0.616 (0.218)	0.192 (0.218)	0.275 (0.219)	0.386 (0.218)	0.384 (0.218)	0.200 (0.218)	0.432 (1.176)	0.291 (1.178)	0.265 (1.178)	0.370 (1.177)	0.336 (1.178)	0.200 (0.218)
LTA	0.460^*^ (0.236)	0.419^*^ (0.237)	0.443^*^ (0.237)	0.456^*^ (0.237)	0.464^*^ (0.237)	0.468^**^ (0.237)	0.109 (0.128)	0.107 (0.128)	0.851 (0.0128)	0.937 (0.128)	0.103 (0.128)	0.468^**^ (0.237)
DIV	0.0124^***^ (0.937)	0.125^***^ (0.938)	0.125^***^ (0.939)	0.125^***^ (0.939)	0.0126^***^ (0.939)	0.126^***^ (0.938)	0.690^***^ (0.506)	0.697^***^(0.506)	0.689^***^ (0.507)	0.688^***^ (0.507)	0.692^***^ (0.507)	0.126^***^ (0.938)

CON	0.440 (0.324)	0.916^***^ (0.320)	0.107^***^ (0.315)	0.114^***^ (0.312)	0.114^***^ (0.312)	0.116^***^ (0.316)	0.566^***^ (0.172)	0.934^***^ (0.169)	0.936^***^ (0.167)	0.962^***^ (0.165)	0.955^***^(0.165)	0.116^***^ (0.316)

GDPpc	0.631 (0.714)	0.145^**^ (0.713)	0.157^**^ (0.726)	0.151^**^ (0.712)	0.148^**^ (0.712)	0.261^***^ (0.749)	0.195^***^ (0.378)	0.224^***^ (0.378)	0.217^***^ (0.385)	0.250^***^ (0.377)	0.240^***^ (0.377)	0.261^***^ (0.749)
INF	−0.168^**^ (0.772)	−0.195^**^ (0.783)	−0.219^***^ (0.768)	−0.218^***^ (0.772)	−0.236^***^ (0.769)	−0.158^**^ (0.786)	0.560 (0.410)	0.220 (0.416)	0.149 (0.407)	0.390 (0.410)	0.961 (0.408)	−0.158^**^ (0.786)

GEF	0.898^***^ (0.136)						6.075^***^ (0.720)					
										
GEF^*^COVID	0.174^***^ (0.366)						0.477^***^ (0.163)					
										
PST		0.213^**^ (0.850)						0.640^***^ (0.211)				
										
PST^*^COVID-19		0.152^***^ (0.394)						0.640^***^ (0.211)				
										
RQL			0.309^***^ (0.031)						0.821^***^ (0.204)			
										
RQL^*^COVID-19			0.124^***^ (0.381)						0.821^***^ (0.204)			
										
COC				0.394^**^ (0.155)						3.991^***^ (0.829)		
										
COC^*^COVID-19				0.149^***^ (0.306)						0.477^***^ (0.163)		
										
ROL					0.461^***^ (0.004)						3.175^***^	
											(1.089)	
ROL^*^COVID-19					0.143^***^ (0.325)						0.581^***^ (0.174)	
VOA						0.683^**^ (0.318)						0.269^***^ (0.063)
VOA^*^COVID-19						0.178^***^ (0.317)							0.178^***^ (0.0317)
$\alpha_{{}_{0}}$	−5.451^***^ (0.598)	−5.026^***^ (0.603)	−5.265^***^ (0.603)	−5.283^***^ (0.598)	−5.235^***^ (0.598)		−5.239^***^ (0.600)	−9.413^***^ (0.246)	−9.005^***^ (0.275)	−8.126^***^ (0.275)	−9.875^***^ (0.251)	−9.967^***^ (0.250)		−5.239^***^ (0.600)
Observations	2,345	2,345	2,345	2,345	2,345	2,345	2,345	2,345	2,345	2,345	2,345	2,345
R-squared	0.054	0.052	0.052	0.052	0.052	0.052	0.045	0.042	0.043	0.043	0.043	0.052
Bank FE	Yes	Yes	Yes	Yes	Yes	Yes	Yes	Yes	Yes	Yes	Yes	Yes
Time FE	Yes	Yes	Yes	Yes	Yes	Yes	Yes	Yes	Yes	Yes	Yes	Yes

The role of the institutional quality. This table presents the country’s institutional quality role during the COVID-19 pandemic. The sample comprises 1,575 banks in 85 countries from 2020 Q1 to 2021 Q4. Our dependent variable is bank performance measured as ROA and ROE. COVID-19 is our primary explanatory variable of interest, measured as the total number of COVID-19 confirmed cases per million in the country. We used the Worldwide Governance Indicators (WGI) to capture the institutional quality, which contains six different aspects of institutional quality such as government effectiveness (GEF), political stability (PST), regulatory quality (RQL), control of corruption (COC), the rule of law (RUL), and voice and accountability (VOA). Robust standard errors are reported in parentheses.

*** *p* < 0.01.

** *p* < 0.05.

* *p* < 0.1.

### COVID-19 and bank performance: Role of financial development

Additionally, prior empirical and theoretical literature highlight that the development of the financial sector has a positive influence on economic activity by increasing the performance of financial services, capital allocation, technological innovation, the efficiency of resource distribution, risk management, and reducing the risk of crises (Levine, 1997; Vithessonthi and Tongurai, 2016). Therefore, we further examine whether the financial development of a country’s banking system mitigates the pandemic’s adverse effect on bank performance. For this reason, we use the financial development index (FDI) from IMF, which summarizes how developed the financial institution is in terms of its depth (FID), access (FIA), and efficiency (FIE). The finding is reported in Table 6, which shows that the coefficient of COVID-19 remains significantly negative in ROAA and ROAE. At the same time, the coefficient for all financial development measures and their interaction terms are significantly positive with ROAA and ROAE. These findings consistently show that banks in countries with more financial development are less vulnerable to COVID-19 shocks on bank performance than in other countries.

**Table 6:** Bank performance during the COVID-19 pandemic The role of financial development.

	(1)	(2)	(3)	(4)	(5)	(6)	(7)	(8)
Variables	ROAA	ROAA	ROAA	ROAA	ROAE	ROAE	ROAE	ROAE
COVID-19	−0.623^***^	−0.376^***^	−0.537^***^	0.886^***^	−1.905^***^	−2.346^***^	−1.206^**^	3.852^**^
	(0.106)	(0.103)	(0.092)	(0.298)	(0.562)	(0.547)	(0.490)	(1.575)
SIZ	0.544^***^	0.597^***^	0.521^***^	0.551^***^	3.364^***^	4.005^***^	3.376^***^	3.647^***^
	(0.066)	(0.068)	(0.066)	(0.066)	(0.362)	(0.369)	(0.361)	(0.361)
CAP	0.078^***^	0.08^***^	0.078^***^	0.078^***^	0.221^***^	0.246^***^	0.224^***^	0.226^***^
	(0.003)	(0.003)	(0.003)	(0.003)	(0.019)	(0.019)	(0.019)	(0.019)
LA	0.049	0.068	0.089	0.119	0.401	0.835	0.903	1.260
	(0.219)	(0.219)	(0.219)	(0.219)	(1.179)	(1.178)	(1.182)	(1.177)
LTA	0.004^*^	0.004^*^	0.005^**^	0.005^**^	0.011	0.015	0.019	0.021^*^
	(0.002)	(0.002)	(0.002)	(0.002)	(0.012)	(0.012)	(0.012)	(0.012)
DIV	0.012^***^	0.012^***^	0.012^***^	0.012^***^	0.070^***^	0.069^***^	0.070^***^	0.068^***^
	(0.000)	(0.000)	(0.000)	(0.000)	(0.005)	(0.005)	(0.005)	(0.005)
CON	0.008^***^	0.005^*^	0.010^***^	0.012^***^	0.090^***^	0.055^***^	0.089^***^	0.110^***^
	(0.003)	(0.003)	(0.003)	(0.003)	(0.017)	(0.017)	(0.016)	(0.016)
GDPpc	0.014^**^	0.007	0.023^***^	0.016^**^	0.246^***^	0.219^***^	0.275^***^	0.224^***^
	(0.007)	(0.007)	(0.007)	(0.007)	(0.037)	(0.038)	(0.038)	(0.038)
INF	−0.020^***^	−0.022^***^	−0.016^**^	−0.017^**^	0.016	0.005	0.051	0.042
	(0.007)	(0.007)	(0.007)	(0.007)	(0.041)	(0.040)	(0.041)	(0.040)
FDI	0.299^***^				1.253^***^			
	(0.707)				(0.123)			
FDI^*^COVID	0.529^***^				0.947^***^			
	(0.128)				(0.221)			
FID		3.963^***^				0.318^***^		
		(0.723)				(0.070)		
FID^*^COVID-19		0.649^*^				0.703^**^		
		(0.333)				(0.334)		
FIA			0.776^**^				0.671^*^	
			(0.377)				(0.380)	
FIA^*^COVID-19			0.652^***^				0.167^**^	
			(0.103)				(0.071)	
FIE				1.309^***^				0.471^***^
				(0.274)				(0.071)
FIE^*^COVID-19				1.938^***^				1.340^**^
				(0.409)				(0.622)
$\alpha_{{}_{0}}$	−4.779^***^	−3.405^***^	−3.680^***^	−6.501^***^	−8.236^***^	−10.611^***^	−8.200^***^	−4.45^***^
	(0.769)	(0.691)	(0.689)	(0.640)	(0.009)	(0.725)	(0.005)	(0.117)
								
Observations	2,343	2,343	2,343	2,343	2,343	2,343	2,343	2,343
R-squared	0.052	0.052	0.053	0.053	0.042	0.045	0.043	0.048
Bank FE	Yes	Yes	Yes	Yes	Yes	Yes	Yes	Yes
Time FE	Yes	Yes	Yes	Yes	Yes	Yes	Yes	Yes

The role of financial development. This table reports the role of financial development during the COVID-19 pandemic. The sample comprises 1,575 banks in 85 countries from 2020 Q1 to 2021 Q4. Our dependent variable is bank performance measured as ROA and ROE. COVID-19 is our primary explanatory variable of interest, measured as the total number of COVID-19 confirmed cases per million in the country. We have taken the four different indexes from IMF to capture the financial development of a country, such as financial development index (FDI), financial institution depth (FID), financial institution access (FIA), and financial institution efficiency (FIE). Robust standard errors are reported in parentheses.

*** *p* < 0.01.

** *p* < 0.05.

* *p* < 0.1.

## Robustness checks

### Alternative dependent variable

It is challenging to evaluate and capture a bank’s overall performance using a single measure (Lee et al., 2014; Baselga-Pascual and Vähämaa, 2021). Therefore, as robustness, we further examine whether our main findings hold when we use alternative measures of bank performance. For this purpose, we followed existing literature (Liang et al., 2013; Adesina, 2021; Dang and Huynh, 2022) and used the net interest margin ratio (NIM), cost-to-income ratio (CIN), and non-performing loans (NPL) as an alternative measure of for bank performance. The results are reported in Table 7, which shows that COVID-19 coefficients remain statistically significant with a negative (positive) sign in NIM (CIN and NPL) of bank performance measures.^3^ This finding shows an adverse impact of COVID-19 on bank performance remains consistent with the previous findings in Table 3.

**Table 7:** Alternative dependent variable.

	(1)	(2)	(3)
Variables	NIM	CIN	NPL
COVID-19	−0.585^***^	1.666^**^	0.148^***^
	(0.064)	(0.808)	(0.003)
SIZ	0.165^***^	−11.85^***^	−0.170^***^
	(0.051)	(0.652)	(0.036)
CAP	0.092^***^	−0.202^***^	0.006^**^
	(0.002)	(0.034)	(0.002)
LA	−0.377^**^	−11.22^***^	−0.155
	(0.173)	(2.158)	(0.118)
LTA	−0.002	−0.242^***^	−0.018^***^
	(0.001)	(0.023)	(0.001)
DIV	−0.040^***^	−0.203^***^	−0.001^***^
	(0.000)	(0.009)	(0.000)
CON	−0.010^***^	0.152^***^	0.001
	(0.002)	(0.030)	(0.002)
GDPpc	0.004	−0.174^**^	0.006^**^
	(0.005)	(0.069)	(0.002)
INF	0.032^***^	−0.356^***^	−0.002
	(0.005)	(0.074)	(0.003)
$\alpha_{{}_{0}}$	4.035^***^	173.8^***^	3.459^***^
	(0.469)	(5.870)	(0.373)
Observations	2,388	2,388	1,155
*R*-squared	0.191	0.042	0.026
Bank FE	Yes	Yes	Yes
Time FE	Yes	Yes	Yes

This table presents the result of our baseline regression models by using the net interest margin ratio (NIM), cost-to-income ratio (CIN), and non-performing loans (NPL) as alternative measures of bank performance. The sample comprises 1,575 banks in 85 countries from 2020 Q1 to 2021 Q4. COVID-19 is our primary explanatory variable of interest, measured as the total number of COVID-19 confirmed cases per million in the country. Robust standard errors are reported in parentheses.

*** *p* < 0.01.

** *p* < 0.05.

* *p* < 0.1.

### Alternative methodology

Our model may have possible endogeneity issues due to reverse causality, omitted variable, and control variable. Therefore, we employ the generalized method of moments (GMMs) and use the two-step system estimator with adjusted standard error for potential heteroskedasticity proposed by Blundell and Bond (1998) as robustness to test our main outcomes are sensitive to estimation approaches. The technique accounts for the unobserved heterogeneity and the dynamic nature of panel data. Moreover, it is more appropriate to deal with possible endogeneity issues and is highly reliable even in reverse causality, omitted variables, and measurement errors (Bond and Hoeffler, 2001). Table 8 describes the results of the System GMM. We find that our baseline finding in Table 3 is still consistent even though we are considering unobserved heterogeneity, simultaneity, and dynamic endogeneity.

**Table 8:** Alternative methodology.

	(1)	(2)	(3)	(4)	(5)
ROAA	ROAE	NIM	EFF	NPL
L.BP	0.198^***^	0.456^***^	0.291^***^	0.664^*^	0.455^***^
	(0.071)	(0.036)	(0.053)	(0.380)	(0.073)
COVID-19	−0.165^**^	−0.027^***^	−0.076^*^	0.013^***^	0.339^***^
	(0.058)	(0.007)	(0.041)	(0.001)	(0.071)
SIZE	0.287^***^	1.683^*^	0.094	−6.219^***^	0.214^***^
	(0.106)	(0.972)	(0.166)	(1.654)	(0.070)
CAP	0.059^***^	0.134^***^	0.031^***^	0.068	0.180^**^
	(0.008)	(0.044)	(0.007)	(0.088)	(0.071)
LIQ	0.159	0.173	−0.078	−2.794	0.049
	(0.262)	(1.479)	(0.222)	(4.617)	(0.119)
LTA	0.008^**^	0.013	0.003	−0.101^*^	0.345^***^
	(0.004)	(0.019)	(0.003)	(0.055)	(0.071)
DIV	0.008^***^	0.041^***^	−0.012^***^	−0.147^***^	0.226^***^
	(0.002)	(0.010)	(0.002)	(0.042)	(0.069)
CON	0.002	0.022	0.016^***^	0.081^**^	0.141
	(0.004)	(0.022)	(0.003)	(0.041)	(0.118)
GDPpc	0.008	0.121^***^	0.001	−0.098	0.673^***^
	(0.006)	(0.034)	(0.005)	(0.075)	(0.070)
INF	−0.001	0.029	0.008	−0.225^***^	0.130^*^
	(0.008)	(0.051)	(0.007)	(0.086)	(0.071)
$\alpha_{{}_{0}}$	−3.382^***^	−15.508^*^	0.636	83.438^***^	−0.405
	(1.028)	(8.735)	(1.478)	(13.945)	(0.492)
Observations	2,273	2,273	1,883	1,883	1,566
AR(1)	0.000	0.000	0.000	0.000	0.000
AR(2)	0.181	0.271	0.268	0.151	0.361
Hansen	0.362	0.526	0.218	0.281	0.245

This table expresses the impact of the COVID-19 epidemic on bank performance by using the System GMM. The sample comprises 1,575 banks in 85 countries from 2020 Q1 to 2021 Q4. Our dependent variable is bank performance measured as ROA and ROE. COVID-19 is our primary explanatory variable of interest, measured as the total number of COVID-19 confirmed cases per million in the country. Robust standard errors are reported in parentheses.

*** *p* < 0.01.

** *p* < 0.05.

* *p* < 0.1.

## Conclusion

Coronavirus is not just a global epidemic and public health crisis. There is a widespread consensus among economists that this has devastatingly impacted the global economy. The economic damage led by the COVID-19 epidemics is mainly due to the reductions in income, productivity, unemployment increase, and trade disruptions. This study investigates how the COVID-19 outbreak affects the banking sector’s performance worldwide. Our sample comprises 1,575 listed and unlisted banks in 85 countries from 2020Q1 to 2021Q4. We use numerous alternative bank performance measures for a comprehensive examination and robustness. The findings illustrate that the outbreak of COVID-19 has significantly reduced bank performance.

We also determine whether the COVID-19 epidemic’s influence on the bank’s performance depends on the bank’s and country-specific aspects. For this reason, we find that bank performance is most negatively affected by the COVID-19 outbreak in smaller, undercapitalized, and less diversified banks. Moreover, we find a better institutional environment and financial development diminish the negative effects of COVID-19 on banks’ performance. Our primary results continue across alternative model specifications (i.e., GMM). The current study’s findings have important policy implications for researchers, policymakers, regulators, and financial institutions to manage risks within and across countries. As policy implications, the study suggests that the government should provide larger economic support, loosened capital requirements, and adjust insolvency rules to mitigate the negative impact of COVID-19. The current study’s major limitation is related to the small number of banks in our sample. We use data from only 1,575 banks whose quarterly data are available. Therefore, if our sample size were larger, more favorable results may have emerged. In future research directions, this study can be further expanded by comparing the impact of COVID-19 on Islamic versus conventional banks.

## Data availability statement

Publicly available datasets were analyzed in this study. This data can be found at: Bankscope Database, World Governance Indicators, and World Development Indicators.

## Author contributions

All authors listed have made a substantial, direct, and intellectual contribution to the work and approved it for publication.

## Funding

This work was sponsored by the Natural Science Foundation of Shandong Province, China (grant number: ZR2021MG004) and the Youth Entrepreneurship Talent Introduction and Education Team of Colleges and Universities in Shandong Province, China. 

## Conflict of interest

The authors declare that the research was conducted in the absence of any commercial or financial relationships that could be construed as a potential conflict of interest.

## Publisher’s note

All claims expressed in this article are solely those of the authors and do not necessarily represent those of their affiliated organizations, or those of the publisher, the editors and the reviewers. Any product that may be evaluated in this article, or claim that may be made by its manufacturer, is not guaranteed or endorsed by the publisher.

## Footnotes

^1^We choose this sample of banks because of the quarterly availability of data on the Bankscope database.

^2^Hausman test suggests that the fixed-effects estimator is more appropriate compared to the random-effects estimator in our study.

^3^Note that in Table 7, the bank performance measure variables such as cost-to-income ratio (CIN), and non-performing loans (NPL) are calculated in such a way that increases the variables indicates lower bank performance and increase the risk. In the interest margin ratio (NIM) case, a higher value shows more bank profitability.
